# Toll Like Receptors as Sensors of the Tumor Microbial Dysbiosis: Implications in Cancer Progression

**DOI:** 10.3389/fcell.2021.732192

**Published:** 2021-09-17

**Authors:** Valentino Le Noci, Giancarla Bernardo, Francesca Bianchi, Elda Tagliabue, Michele Sommariva, Lucia Sfondrini

**Affiliations:** ^1^Dipartimento di Scienze Biomediche per la Salute, Università degli Studi di Milano, Milan, Italy; ^2^U.O. Laboratorio di Morfologia Umana Applicata, IRCCS Policlinico San Donato, Milan, Italy; ^3^Molecular Targeting Unit, Fondazione IRCCS Istituto Nazionale dei Tumori, Milan, Italy

**Keywords:** toll-like receptor (TLR), cancer, microbiota, tolerance, dysbiosis

## Abstract

The microbiota is a complex ecosystem of active microorganisms resident in the body of mammals. Although the majority of these microorganisms resides in the distal gastrointestinal tract, high-throughput DNA sequencing technology has made possible to understand that several other tissues of the human body host their own microbiota, even those once considered sterile, such as lung tissue. These bacterial communities have important functions in maintaining a healthy body state, preserving symbiosis with the host immune system, which generates protective responses against pathogens and regulatory pathways that sustain the tolerance to commensal microbes. Toll-like receptors (TLRs) are critical in sensing the microbiota, maintaining the tolerance or triggering an immune response through the direct recognition of ligands derived from commensal microbiota or pathogenic microbes. Lately, it has been highlighted that the resident microbiota influences the initiation and development of cancer and its response to therapies and that specific changes in the number and distribution of taxa correlate with the existence of cancers in various tissues. However, the knowledge of functional activity and the meaning of microbiome changes remain limited. This review summarizes the current findings on the function of TLRs as sensors of the microbiota and highlighted their modulation as a reflection of tumor-associated changes in commensal microbiota. The data available to date suggest that commensal “onco-microbes” might be able to break the tolerance of TLRs and become complicit in cancer by sustaining its growth.

## Introduction

All mammals harbor widely diverse active microbial communities, collectively termed the microbiota (Sender et al., 2016). The overwhelming majority of these microorganisms inhabits the distal alimentary tract—primarily the gut. Mutualistic microbes, comprising over 10 trillion microbial cells, inhabit the human gastrointestinal (GI) tract and generate functional metabolites that regulate immunity and host metabolism (O’Hara and Shanahan, 2006). Over the past two decades, gut microbiota-host interactions have been widely studied, leading to the comprehension that it plays a role in health. Thus, significant efforts have been made to characterize the entire human microbiome, and the advent of high-throughput DNA sequencing technology, specifically the targeting of bacterial and archeal 16S ribosomal RNA, has made possible to understand that several other tissues of the human body host their own microbiota and that even sites once thought to be sterile have been reported to contain indigenous microbiota populations.

Besides the gut, a microbial community has been found to be present in other body districts, such as the oral cavity, the nasal passage, the genitourinary tract, the skin, the lungs, and the breast.

For example, the **oral cavity** houses over 700 microorganism species, interacting with themselves and with host cells, governed by several signaling pathways (Lu et al., 2019). There is a lot of variability among individuals, probably due to nutrition, hygiene, and genetic conditions that favor establishment and predominance of each different oral microbiota.

In human, the nasal passage also hosts microbes that have recently been reported to support a healthy microenvironment by altering the resistance to pathogens and immune responses (Hardy et al., 2019).

The flora colonized by the **genitourinary tract** mainly consists of lactic acid-producing microorganisms, such as *Lactobacilli*, whose reduction can lead to bacterial vaginosis (Thomason et al., 1991). Further, non-lactobacillary microbiota can inhabit the genitourinary tract in healthy women, the rate of which varies by ethnicity (Buchta, 2018).

The **skin microbiota**—the body of microorganisms that inhabit the skin—underpins the physiology of and immunity in the skin, rendering it critical for protection by the skin in human by stimulating immune responses or preventing pathogenic microorganisms from colonizing it (Di Domizio et al., 2016).

Some years ago, it was clearly demonstrated that the lung, believed to be sterile until then, was colonized by various microbial populations (Wang et al., 2017). The **lung microbiota** has been shown to promote immune tolerance by limiting inflammatory reactions to particulates that are inhaled (Sommariva et al., 2020).

Recent studies have also highlighted that even **breast tissue** harbors a specific microbiota that differs from communities in other areas. In this tissue, the higher abundance of *Proteobacteria* and *Firmicutes* versus other taxa might result from the host’s attempt to adapt to a fatty acid environment through microbial responses in tissue (Urbaniak et al., 2014).

All these body districts’ associated microbes have important functions in maintaining a healthy body state; therefore, they are a guarantee of healthy immunity. Consequently, the host has developed mechanisms to maintain this symbiosis with the microbiota. Thus, the tolerance to harmless antigens entails protective reactions to pathogens and several regulatory pathways.

In the last years, it has been clearly revealed that the microbes that reside in and on the body influence the initiation and development of cancer and its response to therapies. Moreover, in parallel with the discovery that many tissues host a microbiome, specific changes in resident bacteria have recently been linked to cancer in various tissues. Indeed, changes in the alpha diversity [the number (richness) and distribution (evenness) of taxa that are expected in a sample] were detected in cancerous tissue versus normal tissue, but knowledge of their functional activity and the meaning of their changes remain limited.

Toll-like receptors (TLRs) are central in the host’s ability to detect commensal microbiota or pathogens. Through the direct recognition of ligands derived from commensal organisms and pathogens, TLRs preserve tolerance or initiate an immune response. However, changes in the abundance and composition of the local microbiota in tumors have been associated with altered levels of TLR expression and with their activation.

This review summarizes the current findings on the function of TLRs as sensors of microbiota and focus on their modulation as a mirror of tumor-associated microbiota perturbation.

## Toll-Like Receptors as Sensors of Microbes

Toll-like receptors are pattern-recognition receptors (PRRs) that primarily act as sensors of microbes and are crucial for the development of inflammatory and immune reactions. The expression profile of TLRs is wide, from immune cells, including B cells, macrophages, dendritic cells (DCs), and neutrophils, to non-immune cells, like fibroblasts, keratinocytes, and epithelial cells (Hemmi and Akira, 2005; Table 1).

**TABLE 1:** TLR expression on immune/non-immune cells and their ligands.

**TLRs**	**TLRs expression**	**Ligands**	**Origin of ligands**
TLR1	Monocytes, macrophages, B and T cells, DCs, NK, and non-immune cells (fibroblast, astrocytes, epithelial cells, and keratinocytes)	Tri-acyl lipopeptide	Bacteria and mycobacteria *Neisseria meningitidis*
TLR2	Monocytes, macrophages, DCs, and non-immune cells (fibroblast, astrocytes, epithelial cells, and keratinocytes)	Peptidoglycan and LTA Lipoprotein/lipopeptides A phenol-soluble modulin Glycoinositolphopsholipids Glycolipids Porins Atypical LPS	Gram positive bacteria Pathogens *Staphylococcus epidermidis Trypanosoma cruzi Treponema maltophilum Neisseria Leptospira interrogans* and *Porphyromonas gingivalis*
TLR3	DCs, macrophages, mast cells, NK, and non-immune cells (fibroblast, astrocytes, epithelial cells, and keratinocytes)	Double-stranded RNA	Virus
TLR4	Monocytes, macrophages, DCs, and non-immune cells (fibroblast, astrocytes, epithelial cells, and keratinocytes)	LPS Hsp60	Gram-negative bacteria *Chlamydia Pneumoniae*
TLR5	Monocytes, macrophages, T cells, DCs, and non-immune cells (fibroblast, astrocytes, epithelial cells, and keratinocytes)	Flagellin	Flagellated bacteria
TLR6	Monocytes, macrophages, B and T cells, DCs, NK, and non-immune cells (fibroblast, astrocytes, epithelial cells, and keratinocytes)	Di-acyl lipopetides	Mycoplasma
TLR7	B cells and plasmacytoid DCs	Single-stranded RNA	Virus
TLR8	Monocytes and myeloid DCs	Single-stranded RNA	Virus
TLR9	B cells and plasmacytoid DCs, GI epithelial cells, and keratinocytes	CpG oligodeoxynucleotides	Bacteria and viruses
TLR10	B cells and plasmacytoid DCs	Not determined	Not determined

*The table shows the expression of the 10 TLRs on immune and non-immune cells, the ligands they recognize and the bacteria associated with them.*

There are 10 TLRs in humans and 13 TLRs in mice. TLRs that occupy the plasma membrane include TLR1, TLR2, TLR4–6, and TLR11; TLRs that are found in endosomes, such as TLR3 and TLR7–9, detect nucleic acids.

Of the former, TLR2 recognizes a broad range of pathogen-associated molecular patterns (PAMPs) from bacteria, viruses, fungi and parasites. TLR2 primarily associates with TLR1 or TLR6 to form functionally distinct heterodimers. TLR2-TLR1 senses triacylated lipopeptides that are derived from gram-negative bacteria and mycoplasma; in contrast, TLR2-TLR6 heterodimers become stimulated by diacylated lipopeptides from gram-positive bacteria and mycoplasma.

TLR4, the first such ligand that was reported for this TLR, is activated by bacterial lipopolysaccharide (LPS), a constituent of the outer membrane of gram-negative bacteria. Subsequently, TLR4 has been shown to recognize proteins from mouse mammary tumor virus envelope and respiratory syncytial virus, *Streptococcus pneumonia* pneumolysin and paclitaxel, a cytostatic drug that is derived from plants (Akira et al., 2006). TLR5 senses flagellin, the protein element of bacterial flagella, and thus regulates the development of specific immune cell types. In the small intestine, CD11c^+^CD11b^+^ lamina propria DCs express high levels of TLR5, causing IL-17-producing T helper 17 (TH17) and T helper 1 (TH1) cells to differentiate and immunoglobulin A–producing plasma cells to develop from naive B cells on encountering flagellin (Uematsu et al., 2008).

TLR3 was identified to bind double-stranded RNA (dsRNA). This recognition generates antiviral immune reactions by upregulating inflammatory cytokines and type I interferons. Interestingly, TLR3 activation has been also shown to promote apoptosis in various cell types, including cancer cells (Bianchi et al., 2019, 2020).

TLR7 recognizes RNA virus-derived single-stranded RNA (ssRNA), including that from vesicular stomatitis virus, human immunodeficiency virus and influenza A; synthetic poly(U) RNA; and small interfering (si)RNAs. Based on their high expression of TLR7, plasmacytoid DCs (pDCs) secrete high levels of type I interferon on experiencing a viral infection (Akira et al., 2006), implicating them as sensors of ssRNA viruses. TLR7 on conventional DCs (cDCs) upregulates type I interferon on recognition of RNA species from bacteria, including group B *Streptococcus.*

TLR8 is phylogenetically most similar to TLR7 and, like TLR7, binds to viral ssRNAs and foreign bacteria (Bauer et al., 2008).

TLR9 senses unmethylated 2′-deoxyribo (cytidine-phosphate-guanosine) (CpG) DNA, which abound in bacteria and viruses but not mammalian cells. This response affects the activation of macrophages, DCs, and B cells and propels robust TH1 reactions, prompting TLR9 agonists to be used to generate immune response in several diseases, such as cancer (Le Noci et al., 2015, 2016; Sommariva et al., 2017).

After engagement with their cognate ligands, TLRs promote a response against pathogens through signaling cascades that are triggered when the TLR intracellular domain (TIR) interacts with adaptor molecules, including MyD88, TRIF, TIRAP, or TRAM. Depending on the adaptors, there are two signaling pathways promoted by TLRs: MyD88-dependent and -independent (or TRIF-dependent) pathways. MyD88, the first TIR family member to be reported, is an adaptor of all TLRs except TLR3, upregulating inflammatory cytokine production by stimulating NF-κB and mitogen-activated protein kinases (MAPKs). Conversely, TRIF is stimulated only by TLR3 and TLR4, activating other pathways that stimulate NF-κB and IRF3, resulting in the production of type I interferon and other inflammatory cytokines. TRIF is recruited to TLR4 by TRAM, a sorting adaptor, whereas MyD88 hones to TLR2 and TLR4 through TIRAP. Thus, TLR signaling is MyD88-dependent, inducing inflammatory cytokines, or TRIF-dependent, driving the production of type I interferon and inflammatory cytokines. Interestingly, gut microbial colonization regulates the expression of both MyD88 and TRIF in the intestine, and in turn, they can impact the expression of TLR1, TLR2, TLR4, and TLR5 (Hörmann et al., 2014; Scheeren et al., 2014; Brandão et al., 2015).

Apart from TLRs, PRRs include other classes of receptors that trigger intracellular signaling cascades, which lead to the transcriptional expression of pro-inflammatory cytokines, type I interferon, and other anti-viral proteins that all coordinate the elimination of pathogens and infected cells. They include NOD-like receptors (NLRs) that specifically recognize pathogen-associated molecular patterns (PAMPs) (Wilmanski et al., 2008) and for which, to date, more than 20 members have been discovered; stimulator interferon genes (STING); and endoplasmic reticulum (ER)-localized transmembrane proteins, which are required for the immune response against cytosolic DNA (Taguchi and Mukai, 2019).

Beside their main role in promoting response against pathogens, TLR activation by gut microbiota also contributes to intestinal epithelial renewal. The microbiota interacts with all the cell types in the intestinal crypt (dividing and non-dividing stem cells, Paneth cells), triggering epithelial regeneration and stem cell survival through the sensing of bacterial products by TLR4, Nod1, and Nod2 (Hisamatsu et al., 2003; Neal et al., 2012; Nigro et al., 2014). Additionally, TLR2-dependent proliferation of differentiated epithelial cell in the small intestine has been observed due to an increase in the ERK1/2 and AKT pathways (Göke et al., 1998; Sheng et al., 2003; Hörmann et al., 2014). Thus, microbiota-host interactions can affect the homeostasis and architectural structure of microbes lining tissues.

## TLR-Mediated Discrimination of Commensal Microbiota

The bacterial ligands that are sensed by TLRs are common between whole classes of bacteria and thus are also synthesized by commensal microorganisms (Rakoff-Nahoum et al., 2004).

Thus, the engagement of TLRs also constitutes the primary means by which the host and microbiota communicate to maintain the tolerance against commensals. TLRs are able to discriminate between benign colonization and the presence of pathogens and have developed ways to be either responsive or protective against microbes. This tolerance toward commensals is kept in order to maintain the inflammation rate low, since it might be detrimental for the host, thus maintaining epithelial integrity and homeostasis. Based on recent findings, although the mechanisms that differentiate commensals from pathogens are not fully understood, such pathways have begun to be unveiled. The onset of TLR tolerance as a consequence of microbiota exposure or TLR stimulation is a key aspect of adaptive responses in the fine of balance host and microbiota. Repeated LPS stimulation reduces pro-inflammatory responses in monocytes (Randow et al., 1995; Yoza et al., 2000) and decreases leukocyte binding in endothelial cells (Ogawa et al., 2003). Moreover, the crucial role of regulatory mechanisms of TLR signaling in tuning tolerance was evidenced also in epithelial cells concerning postnatal microbial colonization. Indeed, fetal epithelial cells are able to respond to LPS, whereas after birth, endotoxin exposure induces TLR tolerance in epithelial cells by IL-1 receptor downregulation, to finally facilitate the acquisition of a stable symbiosis (Lotz et al., 2006).

### Discrimination in the Gut

**FIGURE 1:**
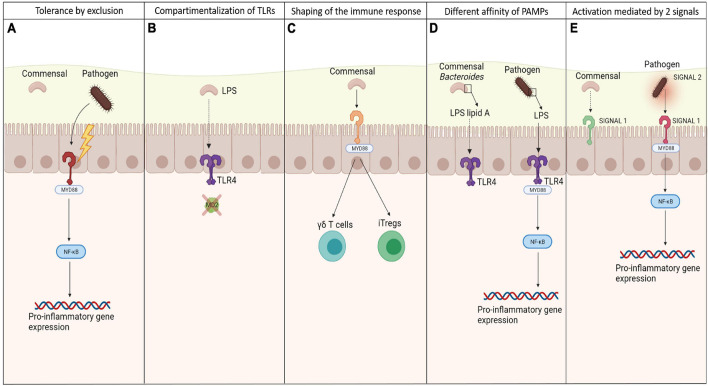
Mechanisms of discrimination between commensals and pathogens by TLRs. Different mechanisms exploited by TLRs to discriminate between pathogenic and commensal microbes to keep the tolerance toward the local microbiota, preventing from inflammation: **(A)** more virulence possessed by pathogens, allowing them to destroy the epithelial barriers and set off an inflammatory state, after being recognized by TLRs; **(B)** specific compartmentalization of some TLRs and their co-receptors, allowing the recognition of bacterial signals only whether they are able to cross the epithelia; **(C)** the ability of commensals in shaping the immune system, maintaining the low inflammation rate and a balance between effector and suppressive cells; **(D)** the different affinity of PAMPs to TLRs so that they are able to recognize only the one relative to pathogens; **(E)** the need of 2 signals to activate an immune response, which come mainly from pathogens causing stress.

Although TLRs are exposed to a significant charge of commensal bacteria, the efficient crosstalk between TLRs and microbiota causes hypo-responsiveness state against the resident microbiota, allowing homeostasis to be maintained. Different strategies have been evolved to regulate the interaction between TLRs and bacterial signals, allowing discrimination between commensals and pathogens (Figure 1). These strategies have been primarily investigated in the gut.

Commensals and pathogens can be differentiated most simply through differences in invasiveness, since constitutive mechanisms physically impede microorganisms from penetrating the host. The intestinal tract is lined by a monolayer of epithelial cells that are joined by tight junctions, which minimizes diffusion. Apically, epithelial cells are lined by microvilli that provide tolerance in the mucosa, generating alkaline phosphatases that detoxify LPS (Srinivasan, 2010) and a thick mucous layer of mucins that reduce the motility of bacteria (Sansonetti, 2004; Johansson et al., 2008). Colonization is also prevented by antimicrobial peptides, including defensins. However, pathogens can disseminate by producing virulence factors that allow them to adhere to and cross epithelial cells.

Commensals that lack virulence factors are ultimately excluded—a property that explains why commensals do not induce inflammation. This process is known as tolerance by exclusion. However, even if apparently excluded, commensal bacteria seem in some way to directly influence the force of the epithelial barriers through the interaction with TLRs. Some studies have revealed that the presence of specific microbial products from commensal bacteria that are recognized and signal through TLRs induces the activation of molecules that favor the intercellular junctions between the epithelial cells, tightening the barriers against pathogens. Notably, MyD88−/− and TLR2−/− mice experience disruptions in tight junctions early in development, increasing their risk of dextran sulfate sodium-induced colitis *versus* wild-type mice (Cario et al., 2007). In corollary findings, these mice also become more permeable to small molecules and have lower transmucosal resistance, enhancing bacterial translocation to the liver, blood, spleen, and mesenteric lymph nodes (Frantz et al., 2012).

The second level of discrimination is based on the expression pattern of some TLRs on epithelial cells, which consists of restricting them to areas that are inaccessible to commensals. The ligand LPS triggers pro-inflammatory signals through TLR4, which acts in concert with two additional LPS-binding proteins: MD-2 and CD14 (Beutler, 2000). Mucosal intestinal epithelial cells have low levels of TLR4 but do not express MD-2 and (Abreu et al., 2001) or maintain the TLR4-MD-2 complex, which is expressed only by mononuclear cells in the lamina propria and crypt epithelial cells (Ortega-Cava et al., 2003). Thus, this tactical compartmentalization allows commensal LPS hypo-responsiveness. The same result is obtained by compartmentalization of flagellin, the major structural subunit of bacterial flagella, binding TLR5 receptor. This PRR is confined to the basolateral surface of epithelial cells and is detected only when the epithelial barrier is breached by a pathogen.

The third level of discrimination is based on the ability of TLR-commensal signaling in shaping the immune response. Intra-epithelial γδ T cells have recently been implicated in the maintenance of homeostasis. These cells abound between intestinal epithelial cells. When the mucosa is disrupted, γδ T cells produce cytoprotective factors, including keratinocyte growth factor, bactericidal proteins and chemokines. For such secretion to occur, commensals and commensal-mediated tissue damage must be sensed by the TLR-induced MyD88 pathway (Ismail et al., 2009).

Individual species in a microbiota can alter immune cell proportions, indicating that the immune response is influenced by the makeup of the microbiota. One key antigen that drives the ratio between the different T subpopulations is flagellin. This major structural subunit of bacterial flagella is critical for T-effector and iTreg cell balance and function through its interaction with TLR5 on CD4 + T cells (Himmel et al., 2008). For instance, low concentrations of flagellin upregulate Foxp3 and increase the resulting suppressive activities of Tregs; in contrast, high concentrations stimulate T effector function through ligation with TLRs.

Moreover, *Bacteroides fragilis*, a common member of the microbiota, has been demonstrated to modulate Treg function through polysaccharide A (PSA) *via* TLR2 signaling (Round et al., 2011). Through the production of capsular PSA, this commensal bacterium inhibits trinitrobenzene sulfonic acid (TNBS)-induced colitis in mouse.

Differences of affinity of commensals and pathogens PAMPs for their TLRs or the production of different anti-inflammatory signals can be considered a further discriminatory mechanism. For example, some *Bacteroides* species, a common gut commensal, have structurally unique LPS domains, as opposed to other enterobacterial species. This LPS has penta-acylated lipid A and a monophosphorylated disaccharide backbone, resulting in a lower affinity to TLR4 (Weintraub et al., 1985; Mancuso et al., 2005). Variations in the number of acyl chains in lipid A impact TLR4 signaling, dramatically altering the host immune response to the pathogen (Table 2). Indeed, while commensals can express a different domain structure in order not to activate TLRs, many others can use these mechanisms as an immune evasion strategy. *LpxM –* the enzyme involved in the final steps of lipid A biosynthesis together with LpxL in *Escherichia coli* – mutants produce predominantly penta-acylated lipid A (Clementz et al., 1997).

**TABLE 2:** The different lipid A structures expressed by bacteria.

**Bacteria expressing different lipid A structures**	**Lipid A structures**
*Bacteroides fragilis*	Penta – acylated lipid A
*E. coli*	Penta – acylated lipid A
*N. meningitidis*	Penta – acylated lipid A
*Bordetella pertussis*	Penta acylated lipid A
*Y. pestis* (37°C)	Tetra – acylated lipid A
*Helicobacter pylori*	Tri/tetra – acylated lipid A
*Pseudomonas aeruginosa*	Hexa – acylated lipid A

*The table shows some examples of the different variations in the acyl group of LPS lipid A and which bacteria express them.*

*Neisseria meningitidis* expresses a hexa-acylated lipid A (Kulshin et al., 1992), and inactivating mutations in *lpxL1*, the homolog of LpxL, have been found in meningococcal disease isolates of *N. meningitidis* (Fransen et al., 2009) to express penta-acylated lipid A.

*Helicobacter pylori* is a widespread human pathogen that lives in the gastric mucosa (Taylor and Blaser, 1991), expressing the canonical hexa-acyl lipid A structure only in minor amounts; the majority of the species is mono-phosphorylated, decorated with a phosphoethanolamine group on the 1-phosphate, and has only three or four acyl chains (Ogawa et al., 1997).

Changes in acylation also affect TLR4 activation in *Yersinia pestis*, whereby temperature-dependent expression of tetra-acyl lipid A resulted in decreased stimulation of TLR4 (Kawahara et al., 2002). Indeed, at 27°C, *Y. pestis* lipid A is hexa-acylated and modified by an aminoarabinose group on the phosphate group. At 37°C, *Y. pestis* expresses lipid A with only four acyl chains.

Others, such as *Bordetella pertussis*, express normally a penta-acylated lipid A (Caroff et al., 2000). Mutants in lipid A-modifying enzymes consisted mostly of tetra-acylated lipid A (Geurtsen et al., 2006). Also, while *Pseudomonas aeruginosa*, an opportunistic pathogen, was found to express penta-acylated lipid A, isolates from the airways of cystic fibrosis (CF) patients had hexa-acyl lipid A, due to the addition of a palmitate group (Ernst et al., 1999).

Some commensal bacteria have evolved mechanisms to inhibit NF-κB signaling. For example, *Bacteroides thetaiotaomicron*, a commensal anaerobic gut bacterium, mediates transport of peroxisome-proliferation-activated-receptor-γ (PPARγ), which associates with the RelA/p65 subunit of NF-κB, from the nucleus, reducing the receptor’s availability (Kelly et al., 2004; Pédron and Sansonetti, 2008), while *Lactobacillus casei* impedes IkB poly-ubiquitination (Collier-Hyams and Neish, 2005).

Finally, a further level of discrimination is related to the need of two signals to activate an immune response, as stated by the well-known Matzinger’s danger hypothesis. Based on this, the innate immune system is stimulated primarily by “danger” signals from injured or stressed cells that are detected through PRRs (Matzinger, 2002). Signal 1 is represented by TLR agonists, expressed both on commensals and pathogens, and their interaction with TLRs effects NF-Kb-mediated production of pro-IL-1b (Mariathasan and Monack, 2006). Signal 2 is provided by a danger signal solely from virulent pathogens that cause cell stress, such as ATP or bacterial toxins, but not by quiescent commensals. Thus, signal 1 can originate from commensals and pathogenic microbes, whereas virulent pathogenic microbes are the only source of signal 2.

### Mechanisms Described in Other Tissues

Since several other districts beside the gut are populated by commensal microbes, it is reasonable to speculate that similar mechanisms that allow the discrimination between commensal and pathogen bacteria can also occur in other tissues. Indeed, TLRs play a crucial role in sensing microbes also at these peripheral sites.

For instance, in the oral epithelium, inhabited by a dense microbial population, all TLR expression has been observed in the basal cell layers except from TLR5, which showed staining throughout the intermediated layers (Salem et al., 2017). The regulation of TLRs is critical for homeostasis in the oral tissue, due to significant amounts of commensals in the oral cavity. Indeed, oral epithelial cells do not generally produce inflammatory mediators, likely to avoid inflammatory cell recruitment and an excessive innate immune response that could result in tissue damage. It has been demonstrated that TLR2 and TLR4 are key regulators in maintaining neutrophil homeostasis in the oral cavity: TLR2−/− and TLR4−/− mice showed an increase in neutrophil number in the junctional epithelium and changes in the makeup of the oral microbiota, suggesting the involvement of these two receptors in maintaining the balance between commensal bacteria and the host in healthy periodontal tissue (Chang et al., 2019).

The skin epithelium also expresses TLRs to accomplish its function as a barrier to pathogen invasions and infections. Through a strict interplay between its resident microbes, keratinocytes, and resident immune components, host homeostasis is well maintained in the skin.

Evidence provided by Pivarcsi et al. (2003) shows that keratinocytes express functional TLRs, particularly TLR2 and TLR4. In a study conducted by Lai et al. (2009) keratinocytes have been found to also express TLR3. After injury, the resulting inflammation is dampened by commensal staphylococcal lipoteichoic acids (LTAs) that interact with surface-bound TLR2 on keratinocytes, suppressing TLR3 signaling through induction of the negative regulator TRAF1 (Lai et al., 2009). Overall, these findings show that also in keratinocytes, a sensitive balance exists between pathogens and commensals due to the crosstalk among TLRs.

The airways harbor significant microbial populations that govern protective mechanisms in the airway and local immune reactions, necessitating a tight regulation of TLR expression levels. Resistance to deadly inflammatory responses is mediated by commensal *Staphylococcus aureus* that inhabits the upper respiratory tract (URT) (Wang et al., 2013) through the passage of CCR2^+^CD11b^+^ monocytes into the alveoli from the bloodstream and their development into M2 macrophages—processes that are TLR2-dependent. Conversely, such inflammation protection is negated in the absence of TLR2. Suppression of influenza-induced inflammation by M2 macrophages is mediated by their secretion of anti-inflammatory molecules and expression of immunomodulatory ligands. In separate human studies, *Bacteroidetes* strains have been shown to limit inflammation in the lung (Larsen et al., 2015), whereas *Prevotella* spp. and *Veillonella* spp. exacerbate Th17-mediated pulmonary inflammation (Segal et al., 2016).

Overall, these studies indicate that the discrimination between commensals and pathogens is guaranteed by several different strategies, most of which involve the modulation of expression and activation of TLRs. These mechanisms are crucial in sustaining the immune “tolerance” to the commensal microbiota and to maintain homeostasis.

## The Role of Toll-Like Receptors in Cancer Progression and Treatment

Different studies have demonstrated that TLRs play a very important role in cancer disease (Coussens and Werb, 2002; Karin and Greten, 2005; Kagan et al., 2008; Rakoff-Nahoum and Medzhitov, 2009; Kaczanowska et al., 2013). Within the tumor tissue, cancer cells, the epithelial component and the mesenchymal/myeloid compartment express TLRs, which, in turn, play a dichotomous role in tumor progression with pro-tumor, as well as anti-tumor, effects (Cen et al., 2018).

TLR2 expressed by innate immune cells is essential to detect danger signals released by cancer cells and to induce a specific anti-tumor immune response (Urban-Wojciuk et al., 2019). Conversely, TLR2 expressed by immune cells, such as Tregs, MDSCs and macrophages, promotes an immunosuppressive microenvironment that leads to tumor progression (Shimizu et al., 2018). Functional TLR overexpression has been found in colon cancer, hepatocellular carcinoma, ovarian and cervical cancers, breast and prostate cancers, lung cancer, melanoma and neuroblastoma (Yang et al., 2010).

TLR2 activation on cancer cells may induce cell proliferation and invasion through different cell-intrinsic mechanisms (Kaczanowska et al., 2013; Chen et al., 2018; Liu et al., 2019). For instance, Scheeren et al. (2014) highlighted a cell-intrinsic role in oncogenesis of the TLR2-MYD88 axis in intestinal and breast epithelial cells, where the inhibition of TLR2 (or downstream targets MYD88) reduced the growth of tumor cells. The TLR4-MYD88 signal is also implicated in spontaneous tumorigenesis. In the Apc^*min/+*^ murine model, both the depletion of MyD88 and TLR4 induced a reduction of intestinal tumors (Koliaraki et al., 2019). Michael et al., showed that TLR4/MYD88 signaling promotes tumor growth and contributes to chemo-resistance against paclitaxel in ovarian cancer (Kelly et al., 2006). Moreover, a recent study delineates that high TLR7 and TLR8 expression promotes chemo-resistance, leading to increased tumor cell proliferation in human pancreatic cancer (Grimmig et al., 2015).

Toll-like receptors have been found to be associated with either a good or bad prognosis. In esophageal cancer, TLR9 expression correlates with advanced stage and high proliferation (Kauppila et al., 2011; Sheyhidin et al., 2011). In lung cancer, TLR5 is associated with a good prognosis, while TLR7 is associated with a poor clinical outcome (Gu et al., 2018). In a recent study, we demonstrated that TLR3 expression on tumor cells predicts a favorable outcome in Stage I NSCLC, whereas TLR3 expression on the immune cells infiltrating the tumor stroma was associated with a poor overall survival. Patients with TLR3-positive immune infiltrating cells but not tumor cells showed a worse prognosis compared with all other patients (Bianchi et al., 2019). High TLR7 and TLR8 expression in melanoma is associated with high expression of antitumor immune markers (CCR2, CCR5, and their respective ligands CCL2, CCL3, CCL4, and CCL5, which induce a chemoattracting microenvironment, which results in the recruitment of immune cells to the tumor site) and predicts longer overall survival (Zhang et al., 2017). In bladder cancer, TLR4 is a favorable prognostic gene to predict overall survival and cancer-specific survival rate (Lu et al., 2021). TLR9 expression in renal cell carcinoma is associated with better survival (Ronkainen et al., 2011). In breast cancer, low tumor TLR9 expression predicts shorter disease-free survival in triple-negative breast cancer patients (Tuomela et al., 2012). TLR9 expression in glioma is associated with poorer survival (Wang et al., 2010). In prostate cancer, TLR9 expression is associated with decreased progression-free survival (Väisänen et al., 2013). Lastly, in a meta-analysis including 15 studies on solid tumors (breast cancer, hepatocellular carcinoma, epithelial ovarian cancer, colorectal cancer, oral squamous cell carcinoma, non-small-cell lung cancer, and pancreatic ductal adenocarcinoma), elevated expression of TLR4 was associated with poor overall survival and shorter disease-free survival (Hao et al., 2018).

In the context of cancer therapy, TLR agonists were found to play an important role in the activation of the immune system. Indeed, in *in vivo* models, TLR agonists have been shown to reduce tumor growth alone, in combination with chemotherapy drugs or with monoclonal antibodies (Davis et al., 2011; Triozzi et al., 2011). Bacillus Calmette-Guèrin (BCG) (a TLR2/4 ligand) with imiquimod (a TLR7 agonist) and monophosphoryl lipid A (MPLA, a TLR4 ligand) are the three TLR ligands approved by the FDA for cancer treatment. BCG reduced recurrence and prolonged survival in bladder cancer patients (Morales et al., 1976). MPLA resulted in a potent vaccine adjuvant and promoted type 1 T helper immune responses in cervical cancer induced by human papillomavirus (Dhodapkar et al., 2014). Imiquimod induced apoptosis and stimulated cell-mediated immune responses in various cutaneous malignancies (Adamus and Kortylewski, 2018); lastly, CpG-ODN (a TLR9 agonist) enhanced the efficacy of immune checkpoint inhibitors in several types of cancer (Adamus and Kortylewski, 2018).

Furthermore, in two different studies, we have demonstrated that the TLR agonists are effective in increasing the antitumor activity of chemotherapeutic and immunotherapic models in preclinical studies. Specifically, the combination of CpG-ODN and Poly(I:C)—TLR9 and TLR3 agonists, respectively—with dacarbazine led to a significant increase in the inhibition of B16 melanoma lung metastases (Le Noci et al., 2015), and the antitumor activity of TLR9 and TLR3 agonists was improved by combination with the antibody anti-MDSC and INFα-based immunotherapy.

Toll-like receptor ligands have also been shown to be effective as adjuvants with anti-PD-1 therapy. R848, the ligand of TLR7/8, in combination with anti-PD-1 antibody inhibited tumor growth in an *in vivo* model of PD-1-resistant melanoma (Rodell et al., 2018). The TLR3 agonist ARNAX has shown to be capable of overcoming resistance to PD-L1 inhibition in an *in vivo* model of lymphoma, leading to tumor regression (Matsumoto et al., 2017; Takeda et al., 2017).

However, TLR ligands may represent a double-edged sword in the treatment of cancer. TLR4 stimulation by LPS was found to increase the production of immunosuppressive cytokines that contribute to tumor immune escape and induced resistance to apoptosis in lung cancer cells (He et al., 2007). The stimulation of TLR7/8 over-expressed on a pancreatic cancer cell line resulted in increased cell proliferation and reduced chemosensitivity (Grimmig et al., 2015).

## Cancer-Associated Dysbiosis

Emerging evidence indicates that commensal bacteria are also found at tumor sites. A recent study analyzed 1526 tumor specimens, including breast, lung, ovary, pancreas, melanoma, bone, and brain cancers, and adjacent normal tissues. Notably, each tumor type was associated with a unique microbiome pattern (Nejman et al., 2020). The presence of a microbiome in the tumor was found to be extremely variable, depending on tumor histotypes, with breast cancer having a particularly rich and diverse microbiome. Solid tumors that are shielded and internal, such as ovarian and bone cancer, also harbored bacterial DNA. The presence of bacteria in human tumors was confirmed by immunohistochemistry (IHC), staining for LPS and LTA and thus gram-negative and gram-positive bacteria, respectively (Raetz and Whitfield, 2002), and by RNA fluorescence *in situ* hybridization (FISH) (Amann et al., 1990). Moreover, to further validate the presence of live and metabolically active bacteria at the tumor site, slices from fresh breast tumors were cultured *ex vivo* in the presence of fluorescently labeled D-alanine or dimethyl sulfoxide (DMSO) as control (Siegrist et al., 2013), confirming intracellular labeling in all tumor sections.

Interestingly, several studies comparing 16S rRNA profile of normal and tumoral tissue have revealed changes in microbiome abundance and composition in tumors (Kovács et al., 2019). Jin et al. (2019) reported a significant increase in the total bacteria burden, as well as reduced bacterial diversity in the lung tumors compared to healthy lung in mice. In particular, some bacterial taxa, such as *Herbaspirillum* and *Sphingomonodaceae*, were over-represented in lung tumor-bearing mice, and others, such as *Aggregatibacter* and *Lactobacillus*, were enriched in healthy lungs.

On the contrary, in a pilot study conducted by Peters et al. (2019) in human lung tumor samples, a clear reduction in bacterial richness and diversity was observed in lung tumor samples, compared with normal tissues, supporting dysbiosis of the lung tumor microbiome.

In a study by Pushalkar et al. (2018), a markedly more abundant microbiome was harbored by cancerous pancreatic tissue compared with normal pancreas, in both mice and humans. The abundance of the genus *Brevibacterium* and the order *Chlamydiales* was seen in human normal pancreas compared to pancreatic tumor.

*Porphyromonas gingivalis*, a periodontal pathogen that was found to be higher in patients with pancreatic cancer than healthy subjects, was associated with an increased mortality rate (Karpiński, 2019). Differences in the microflora of pancreatic ductal cancer were also reported by Farrell et al. (2012), with significantly lower and higher levels of *Neisseria elongata*, as well as *Streptococcus mitis* and *Granulicatella adiacens*, respectively.

In the breast, changes in microbiota composition may be responsible for promoting cancer progression (Fernández et al., 2018; Nejman et al., 2020). Statistically significant differences of the bacterial profiles have been observed in the comparison of normal adjacent breast cancer tissue with healthy breast tissue from women. Higher abundance of *Enterobacteriaceae* and *Staphylococcus* taxa, which display the ability to induce DNA double-stranded breaks, was found in breast cancer than normal breast (Urbaniak et al., 2016). Hieken et al. (2016) found that the breast microbiome in women with malignant disease was notably different from that of women with benign disease.

Moreover, distinct microbiome profiles have been detected in the ovary, fallopian tubes and cervix between normal and cancerous tissues, and these distinct profiles have been associated with endometrial and ovarian cancers (Brewster et al., 2016; Walther-António et al., 2016). For instance, endometrial cancer is significantly linked to the combination of *Atopobium vaginae* and *Porphyromonas* sp. in the gynecological tract and high vaginal pH.

Patients with CRC have a less diverse microbiome than healthy individuals. *Fusobacterium nucleatum* was found to be increased in tumor compared to normal specimens, as well as *E. coli*. Also, oral bacteria relevant in periodontal disease are enriched in CRC (Song et al., 2020).

There are possible intrinsic and extrinsic factors that may contribute to dysbiosis within the cancerous tissue. Among them, the tumor microenvironment, which is highly hypoxic, facilitates the growth of anaerobic and facultative anaerobic bacteria, such as *Clostridia*. Similarly, necrotic areas of the tumor release chemotactic compounds and may attract bacterial invasion. The leaky vasculature of cancerous tissues also allows bacteria to enter the tumor mass, where the absence or low abundance of immune cells may permit their growth (Syed Khaja et al., 2017).

Considering the role of TLRs in regulating and maintaining the integrity of epithelial barriers, favoring intercellular junctions (Cario et al., 2007; Hanson et al., 2011), an alteration of microbiota perturbation affecting the expression pattern of TLRs on the epithelial cell surface and their activation can increase intestinal permeability (Guo et al., 2013; Lopetuso et al., 2017; Nighot et al., 2017). Such alterations are exploited by either microbes and cancer cells to reach the circulatory and lymphatic stream (Chu et al., 2018) and represent a new field of study for cancer research (Fu et al., 2021).

Notably, it is still not clear whether the perturbation of the normal microbiome in tumor is a consequence of tumor-induced changes of the tissue microenvironment or if the altered microbiota is a causal agent or a complicit actor in cancer disease.

It has been found that in CRC, the depletion of neutrophils, which are highly abundant immune cells in CRC, correlates with increased numbers of bacteria in tumors and proliferation of tumor cells, tumor cell DNA damage, and an inflammatory response mediated by interleukin-17 (IL-17). Administration of antibiotics or a neutralizing antibody against IL-17 to neutrophil-deficient mice resulted in development of less-invasive tumors compared to mice given vehicle (Triner et al., 2019).

Accordingly, several lines of evidence indicate that the microbiome could be involved in cancer development and progression by inducing/promoting malignant transformation and by altering immune system homeostasis/balance at the site of tumor growth (Picardo et al., 2019). Changes in bacteria within the tumor environment can be immunomodulatory.

Some evidence supports an immunostimulatory role for bacteria in the tumor environment. In preclinical models, recognition of bacteria by intratumoral innate immune cells via PRRs can activate pro-inflammatory cytokine production, driving further influx of a variety of immune cells and improving antigen presentation, thereby increasing the antitumor immune function (Kim et al., 2017; Zheng et al., 2017). Also, *Lactococcus* species are able to maintain the cytotoxic activity of natural killer (NK) cells. Others, such as *Bifidobacterium*, *Bacteroides thetaiotaomicron*, and *B. fragilis*, are able to stimulate anticancer immunity (Fulbright et al., 2017). Intratumor bacteria can also alter the expression of ligands and receptors on both immune and cancer cells that are current targets of immunotherapy (Smola, 2017; Panda et al., 2018). Indeed, *Bifidobacterium* has been found to facilitate local anti-CD47 immunotherapy on tumor tissues through the capacity to accumulate within the tumor microenvironment, via the signaling pathway of STING (Shi et al., 2020).

On the other hand, other studies suggest that intratumoral bacteria create a predominantly immunosuppressive microenvironment (Al-Hilu and Al-Shujairi, 2020). They can recruit myeloid-derived suppressor cells (MDSCs) and increase the production of immunosuppressive cytokines or the activation of alternative immune checkpoints, conferring a non-cytolytic response (Thiele Orberg et al., 2017). *Fusobacterium nucleatum* inhibits cytotoxic T lymphocytes and enables tumor progression (Fulbright et al., 2017). It was also demonstrated that intra-tumor bacteria in pancreatic cancer led to T-cell anergy in a TLR-dependent manner (Pushalkar et al., 2018).

Bacteria are also able to recruit other species at the tumor sites, by binding the cell surface motifs on cancer cells or immune cells, leading to downstream oncogenic or immunosuppressive signaling. For example, tumor-coating ETBF has been shown to recruit other bacteria, as well as immune cells, to the tumor site and boosts IL-17-mediated inflammation (Dejea et al., 2018).

We recently reported that commensal microbiota plays a role in maintaining an immunosuppressive microenvironment in the lung and that its manipulation by aerosolized antibiotics and probiotics, decreasing the percentage of Tregs and M2-polarized alveolar macrophages, respectively, reduces immune suppression and promotes an immunosurveillance against B16 melanoma metastases (Le Noci et al., 2018). Moreover, to decipher the pro-tumorigenic effects of microbiota alteration, the microbiota’s role in remote signaling also has to be considered. For example, the microbiota indirectly alters platelet function through hepatic TLR2 signaling (Jäckel et al., 2017) and controls tonic IFN-I signaling in cDCs, poising them for their future functions as key coordinators of adaptive immunity (Schaupp et al., 2020).

## Toll-Like Receptors as Sensors of Local Dysbiosis in Cancer

Perturbations in bacterial abundance and composition in tumors have been associated in several studies to the modulation of TLR levels and activity on tumor or immune infiltrating cells, resulting, in turn, in immunomodulation of the immune environment and/or tumor growth (Table 3).

**TABLE 3:** Studies revealing the activation of TLRs in response to bacterial presence/perturbation in various tumors.

**Tumors**	**Bacterial signals**	**Activated TLRs**	**Effects**	**References**
Lower gastrointestinal tumor	*Helicobacter pylori*	TLR2 upregulation	Proliferation of intestinal epithelial cells	Huang et al., 2007
	*Fusobacterium nucleatum*	TLR2/TLR4	Proliferation of cancer cells Resistance to chemotherapy in CRC	Yu et al., 2017; Sun et al., 2019
	LPS	TLR4	M2 macrophages switch and secretion of cytokines in CRC	Li et al., 2019
Esophageal adenocarcinoma	Gram negative bacteria	TLR4	Inflammation, apoptosis blockage, innate, and adaptive immune responses	Neto et al., 2016
Oral squamous cell carcinoma	LPS	TLR4	Cancer progression and migration Tumor escape	Huang et al., 2005; Kurago et al., 2008; He et al., 2015; Zhang et al., 2019
Pancreatic cancer	Distal microbial dysbiosis	TLR2 and TLR5 upregulation	Immunosuppressive phenotype, T cells anergy and increased tumor growth	Pushalkar et al., 2018
Liver cancer	LPS	TLR4	Hepatocellular carcinoma promotion, proliferation and prevention of apoptosis	Dapito et al., 2012
Lung cancer	Pneumotype supraglottic predominant taxa (SPT)	TLR2/4	Attenuated immune responses of alveolar macrophages	Segal et al., 2016
	*Lactobacillus, Streptococcus*, and *Staphylococcus*	TLR-MyD88 dependent pathways	Expansion of IL-17-producing γδ T cells	Jin et al., 2019
	*Staphylococcus aureus*	TLR2	Recruitment and polarization of CCR2 + CD11B + monocytes into M2 alveolar macrophages	Wang et al., 2013
Breast cancer	Lower number of bacteria	TLR2, 5 and 9	Lower pro-inflammatory cytokines as IL-12A	Xuan et al., 2014
	LPS	TLR4	Metastasis	Beutler, 2000
	*Pseudomonas aeruginosa*	TLR4	Metastasis	Li et al., 2017

*The table shows TLRs activated in different tumors (lower and upper gastrointestinal, pancreas, liver, lungs, and breast) by different bacterial signals, derived from microbial changes at the tumor site (dysbiosis). On the right, references are reported.*

### Lower Gastrointestinal Cancers

Bacteria and cancer are connected through TLR modulation, based on findings in gastrointestinal tumors. Tumorigenesis in the colon can be effected by various bacterial species through two overarching pathways: the stimulation of TLRs on cancer cells and the stimulation of pro-tumorigenic mechanisms in host immune cells (Chen et al., 2017; Krzysiek-Maczka et al., 2019). *Helicobacter pylori* is the most extensively studied bacterial etiological factor in gastric adenocarcinoma and colon cancer, causing tumor onset and progression by upmodulating TLR2 on intestinal epithelial cells, which accelerates their proliferation (Huang et al., 2007). As with *H. pylori*, a poor prognosis in colorectal cancer patients is associated with high intestinal levels of *Fusobacterium nucleatum* (*F. nucleatum*). This anaerobic, gram-negative bacteria stimulates TLR2/TLR4- and E-cadherin-dependent activation of NF-κB and the Wnt pathways in cancer cells, allowing tumors to grow (Sun et al., 2019). *F. nucleatum* might also promote chemotherapeutic resistance in colorectal cancer (CRC) via TLR stimulation on cancer cells (Yu et al., 2017).

Moreover, LPS, binding to TLR4, enhances the polarization of M2 macrophages and their secretion of cytokines, which in turn stimulate the migration and mobility of CRC cells (Li et al., 2019).

### Upper Gastrointestinal Cancers

Bacteria exist in cancers of the upper gastrointestinal tract, including esophageal, biliary, and oral. Further, gram-negative bacteria can stimulate TLRs, potentially causing inflammation and malignancy in esophageal and gastric epithelial cells. TLR4 expression is high in esophageal biopsies from esophageal adenocarcinoma (EAC) patients *versus* healthy controls. Upon TLR4 stimulation, NF-κB translocates to the nucleus and activates target genes that mediate inflammation, the blockade of apoptosis, and innate and adaptive immunity. This finding indicates that gastro-esophageal dysbiosis mediates the development of EACs through LPS–TLR4–NF-κB signaling (Neto et al., 2016).

Studies that have focused on oral squamous cell carcinoma (OSCC) revealed a significant increase in LPS biosynthesis at the tumor site and found that LPS enhances progression and migration (Kurago et al., 2008; He et al., 2015). Moreover, LPS could activate TLR4 signaling in tumor cells and help tumor cells to escape attack from cytotoxic lymphocyte (CTL) and natural killer (NK) cells (Huang et al., 2005; Zhang et al., 2019).

### Gastrointestinal Accessory Organs Cancers

The wide range of studies on the gut microbiome and its relationship with the host under healthy conditions and in disease states is due to the largest reservoir of bacteria in the gastrointestinal tract. It is not the same for other tissues, for which there are recent but fewer findings about the presence of bacteria. However, studies have revealed a link between microbiota dysbiosis and TLR activation also in tumors in other districts, such as the ones affecting accessory organs of the gastrointestinal tract, like the pancreas and liver.

Pancreatic cancer is an aggressive type of cancer for which the therapeutic success and survival rates are low. The pancreas does not have a microbiome; nevertheless, its carcinogenesis can be elicited by distal dysbiotic microbiota (Schwabe and Jobin, 2013) via inflammatory responses, LPS expression and TLR4 stimulation (Zambirinis et al., 2014). Pancreatic cancer has several common risk factors, such as periodontal disease and poor oral hygiene, because such conditions are conducive to the translocation of bacteria to the pancreas through circulation. For example, in a murine pancreatic cancer model, LPS stimulates TLR4 on immune cells in the tumor microenvironment, favoring tumor progression through the NF-κB and MAPK pathways (Ochi et al., 2012).

In murine pancreatic cancer models and human pancreatic tumors, bacteria transit to the pancreas from the gut (Pushalkar et al., 2018). In this study, the microbiome derived from the gut locally reprograms the pancreas immune microenvironment, promoting carcinogenesis by establishing immune tolerance, effected through the inhibition of monocyte differentiation via specific TLRs and T cell anergy. TLR2 and TLR5 expression is higher in intratumoral macrophages, and their stimulation accelerates the growth of tumors (Pushalkar et al., 2018). Thus, it appears that immunosuppression in PDAC tumors is established by bacteria via TLR2 and TLR5 signaling in tumor-associated macrophages. Accordingly, the ablation of the microbiome by antibiotics was demonstrated to decrease myeloid suppressor cells associated with the tumor and allowed increased T cell activity (Pushalkar et al., 2018).

Increased translocation of intestinal bacteria has been also involved in liver malignancy. Chronic liver disease is characterized by bacterial translocation from the gut, promoting hepatic inflammation and fibrosis. In a study published by Dapito et al., small molecules from the microbiome were found to be carcinogenic, altering immune reactions through signaling of the LPS-TLR4 axis was clearly revealed (Dapito et al., 2012). In this study, the progression of HCC—but not its initiation—required TLR4 and the intestinal microbiota, which increased cell division, upregulated the hepatomitogen epiregulin, and inhibited apoptosis. HCC was mitigated by gut sterilization that was limited to the later stages of hepato-carcinogenesis; this finding implicates the intestinal microbiota and TLR4 as targets in the treatment of advanced liver disease.

### Respiratory Tract Cancers

Most lung cancer cases (90%) are attributed to smoking; yet, only 15% of smokers suffer from lung cancer, indicating that other factors influence tumor development. The lung interface is exposed to the environment, and the unique microbiota of the lung, comprising various *Proteobacteria* species, reflects microaspiration of the oral microbiota. Various microorganisms, including *Haemophilus influenzae* and *Streptococcus pneumoniae*, are associated with half of all cases of chronic pulmonary disease; these microbes can induce chronic inflammatory responses associated with tumor progression (Houghton, 2013).

In heavy-smoker lung cancer patients, the tumor contains less *Acinetobacter* and *Acidovorax* and more *Streptococcus* and *Prevotella* than tissue from emphysema patients (Liu et al., 2018). To this end, *Veillonella* and *Megasphaera* are sensitive and specific biomarkers for lung cancer (Lee et al., 2016).

In a study published by Segal et al., an analysis of lung microbiota composition divided healthy subjects in two groups, or pneumotypes, who responded differently to TLR4 ligation. Specifically, subjects with the supraglottic pneumotype (SPT) had a high bacterial load, whose taxa originated primarily from the upper respiratory tract (URT)—these properties correlated with the inability of alveolar macrophages (AMs) to respond to LPS. These taxa included *Prevotella, Rothia*, and *Veillonella*, which stimulate AMs in a MyD88-TLR2/4-dependent manner, downregulating LPS-induced IL-6 and MIP-1a production (Segal et al., 2016). These data suggest that the microbiota governs inflammatory reactions on the surface of pulmonary mucosa. Further, on inoculating mice intra-tracheally with late-stage lung tumors from a separate cohort, Jin et al. (2019) identified several bacterial species, including *Lactobacillus, Streptococcus* and *Staphylococcus*, accompanied by the TLR-MyD88-dependent expansion of IL-17-producing γδ T cells, precipitating tumorigenesis. Similarly, IL-17-producing γδ T cells proliferated in lungs when given TLR ligands locally, including LPS and peptidoglycan.

### Female Cancers

In human, breast cancers have a unique microbiota. Different studies highlighted that the bacterial species in the tumor differ versus in the overlying skin or healthy tissue (Banerjee et al., 2015; Hieken et al., 2016; Meng et al., 2018).

Breast cancer tissue harbors fewer bacteria than adjacent normal tissue and expresses lower amounts of TLR (TLR2, 5 and 9) and pro-inflammatory cytokines, such as IL-12A. These data suggest that alterations of the normal breast microbiome alters inflammatory reactions in carcinogenesis (Xuan et al., 2014). Moreover, a study revealed that activation of the MCF-7 and MDA-MB-231 breast cancer (BC) cell lines by LPS induces metastasis through TLR4 (Beutler, 2000), also suggesting a direct role of TLR activation on tumor cells. *P. aeruginosa* is a potent producer of LPS (Pier, 2007), and its stimulation by TLR4 has also been revealed to promote breast cancer metastases through Akt activation (Li et al., 2017).

## Cancer Microbiota and Response to Therapy

The microbiome can modulate the efficacy of both chemotherapy and radiotherapy responses. Indeed, bacteria can inactivate or activate chemotherapeutic drugs or interfere with the side effects of the therapy. The relationship is reciprocal, as tumor therapy can influence the composition and function of the microbiome (Mikó et al., 2019).

Different studies demonstrated that the treatment responsiveness depends on the gut microbiome both in mouse models (Iida et al., 2013; Viaud et al., 2013; Sivan et al., 2015; Vétizou et al., 2015) and in cancer patients (Gopalakrishnan et al., 2018; Matson et al., 2018; Routy et al., 2018).

In a recent study, it has been reported that after CTLA-4 therapy, the microbial composition of the gut was shifted; *Bacteroidales* and *Burkholderiales* abundance decreased, and *Bacteroides* and *Clostridiales* were enriched. Moreover, *B. fragilis* promoted T helper 1 (Th1) responses and activated dendritic cells through the induction of IL-12. Thus, an improvement in anti-CTLA-4 effectiveness may be partially due to the enrichment of *B. fragilis.* Improved effectiveness of anti-CTLA-4 therapy was also observed in melanoma patients with increased abundance of *Bacteroides*, *Bacteroides thetaiotaomicron*, and *B. fragilis* (Vétizou et al., 2015). Studies on anti-PD-1 or anti-PD-L1 therapy showed similar bacteria-driven differences in tumor outgrowth. In a mouse model of melanoma, increased effectiveness of anti-PD-L1 therapy was associated with enhanced *Bifidobacterium* (*Bifidobacterium longum* and *B. breve*) abundance in the gut and a consequent activation of dendritic cells (Sivan et al., 2015). In metastatic melanoma patients receiving anti-PD-1 and anti-PD-L1 treatment, patients with greater alpha diversity, enriched in *Clostridiales*, *Faecalibacterium*, and *Ruminococcaceae* species and lower in *Bacteroidales*, had longer survival (Gopalakrishnan et al., 2018). In another study in advanced melanoma patients, those who responded to anti-PD-L1 therapy had elevated levels of *Bifidobacterium longum*, *Collinsella aerofaciens*, and *Enterococcus faecium* and also carried *Akkermansia muciniphila* (Matson et al., 2018).

Better survival was also shown in urothelial carcinoma, renal cell carcinoma, or non-small cell lung carcinoma patients undergoing anti-PD-1 treatment who carried elevated levels of *Akkermansia* and *Alistipes* species (Routy et al., 2018).

In addition, there is now evidence that the gut microbiota may shape responses to other forms of cancer therapy. The microbiota in the gut and other sites was shown to influence responses to a range of chemotherapies (Paulos et al., 2007; Iida et al., 2013; Viaud et al., 2013). Beneficial responses to cyclophosphamide were linked to increased intestinal permeability, allowing bacterial translocation, resulting in the maturation of T helper 17 (TH17) cells. In contrast, the response to local CpG oligonucleotide therapies and oxaliplatin was dependent on microbiome-dependent changes in the expression of pro-inflammatory genes and the production of reactive oxygen species by myeloid cells within the tumor microenvironment (Iida et al., 2013). In a study conducted by Chiba et al. (2020) neoadjuvant chemotherapy-treated breast cancer patients showed an increase in the tumor proportion of *Pseudomonas*. Normal mammary gland tissue displays a low proportional abundance (approximately 5%) of *Pseudomonas*, whereas breast tumor tissue contains elevated (approximately 20%) *Pseudomonas*. Neoadjuvant chemotherapy further increased the proportional abundance of *Pseudomonas* to 85%, suggesting that chemotherapy induced preferential growth or survival of these bacteria (Chiba et al., 2020). *P. aeruginosa* secreted factors that may also directly impact breast cancer cell proliferative signaling. *P. aeruginosa* is a potent producer of LPS (Pier, 2007), which was previously shown to promote breast cancer metastases through Akt activation (Li et al., 2017). Chiba et al. (2020) showed that *P. aeruginosa*-conditioned media, at low concentrations (5–10%), stimulates breast cancer cell growth and activates protumorigenic Akt signaling, suggesting that a moderate proportional (20%) of *Pseudomonas* in untreated breast tumors may promote tumor survival and growth.

Also, at distal sites, where bacteria have been found within different cancer types, the resident microbiota can influence the response to therapies. For instance, *F. nucleatum* was found to be abundant in CRC tumorous tissues in patients with recurrence postchemotherapy (Yu et al., 2017). *F. nucleatum* was found to promote chemoresistance by activating autophagy pathway through downregulation of microRNAs (miR-18a and miR4802) (Yu et al., 2017). In another study that investigated the role of intratumor microbiota in CD47-based cancer immunotherapy, Shi et al. (2020) found that colonic *Bifidobacterium* accumulates in tumor sites and facilitates local anti-CD47 treatment via the STING pathway.

Seventy-six percent of human pancreatic ductal adenocarcinoma (PDAC) samples were positive for Gammaproteobacteria that conferred resistance to the chemotherapeutic drug gemcitabine when tested on human colon cancer cell lines (Geller et al., 2017).

In addition to bacteria, intratumor fungi, like *Malassezia globosa*, were found to contribute to tumorigenesis, tumor growth, and gemcitabine resistance in pancreatic cancer (Aykut et al., 2019).

In a different study, *Fusobacterium* was found to confer resistance to chemotherapy in CRC through the activation of TLRs on cancer cells and the subsequent loss of certain microRNAs within the tumor and initiation of autophagy (Yu et al., 2017).

Overall, accumulating evidence highlights that conventional cancer therapies can be modulated by the microbiota and interactions with the host through TLRs. Despite our growing knowledge of microbiota in cancer treatments, a better understanding of the crosstalk between tumor-associated bacteria and TLRs in cancer may form the basis for new cancer treatments. TLR activation through recognition of microbe-associated molecular patterns could be a promising immunotherapy-based treatment.

## Conclusion

Toll-like receptors are the primary sensors of both commensal and pathogenic microbes. Indeed, TLRs are normally able to discriminate between benign colonization and the presence of pathogens and have developed ways to be either responsive or protective against the microbes. Besides the gastrointestinal tract, several tissues of the human body host their own microbiota, and even sites once thought to be sterile clearly contain an indigenous microbiota. The perturbation of microbiota abundance and composition has been observed in several cancerous tissues *versus* the normal counterpart. Ongoing studies point to define if this microbial dysbiosis could be an actor able to play a key role in cancer progression or a passive consequence of the local microenvironment changes, such as the establishment of hypoxia, which facilitates the growth of anaerobic and facultative anaerobic bacteria, or the release of chemotactic compounds from necrotic areas, which promotes bacterial invasion. How these changes are sensed by TLRs is not clear.

The modifications of microbiota observed in various tumors are accompanied by the activation of specific TLRs, resulting in a direct proliferative stimulation of tumor or in a chronic inflammatory condition able to enforce the immunosuppressive microenvironment. Thus, these receptors might sense the microbial perturbation within the tumor microenvironment and, breaking tolerance, become complicit of their effects on tumor growth. Greater insights into the crosstalk between the perturbed microbiota and TLRs in tumors will enable a clinical scenario with new strategies to target TLRs in tumors and interfere with microbiota-induced tumor progression.

## Author Contributions

LS had the idea for the manuscript and supervised the entire work. VL and GB performed the literature search. LS, VL, and GB drafted the manuscript. MS, FB, and ET critically revised the manuscript. All authors read and approved the final manuscript.

## Conflict of Interest

The authors declare that the research was conducted in the absence of any commercial or financial relationships that could be construed as a potential conflict of interest.

## Publisher’s Note

All claims expressed in this article are solely those of the authors and do not necessarily represent those of their affiliated organizations, or those of the publisher, the editors and the reviewers. Any product that may be evaluated in this article, or claim that may be made by its manufacturer, is not guaranteed or endorsed by the publisher.
